# Efficacy of immediate loading compared to conventional loading in implant-supported removable prostheses: a systematic review and meta-analysis

**DOI:** 10.2340/aos.v83.42027

**Published:** 2024-10-01

**Authors:** Zhongke Wang, Sihui Li, Hongmei Chen, Ling Guo

**Affiliations:** The Affiliated Stomatology Hospital of Southwest Medical University, Sichuan, PR China

**Keywords:** Immediate dental implant loading, meta-analysis, dental prosthesis, implant-supported, denture, overlay, denture, partial

## Abstract

**Purpose:**

This systematic review and meta-analysis aimed to evaluate the efficacy of two different loading methods in implant-supported removable prostheses (partial dentures and full-maxillary dentures).

**Methods:**

As of August 2023, three electronic databases and nine oral implant-related journals had been searched. Only randomised controlled trials (RCTs) that compare immediate loading with conventional loading in implant-supported removable prostheses were included. The primary outcome was the implant survival rate. At the same time, a meta-analysis of bone-level changes was performed. Cochrane risk-of-bias tool for randomised trials (RoB 2) was used to evaluate the risk of bias in the included trials.

**Results:**

A total of 16 RCTs were included, including 543 participants with 1595 implants. The included trials compared immediate loading and conventional loading in implant-supported removable prostheses. Regarding implants as the statistical unit, the immediate loading group had a statistically significant lower survival rate (RR = 0.950; 95% confidence interval [CI], (0.926, 0.974); *P* = 0.027; *I*² = 47%). When patients were regarded as statistical units, a statistically significant lower survival rate was also observed in the immediate loading group (RR = 0.929; 95% CI, (0.897, 0.961); *P* = 0.590; *I*² = 0%). When we analysed the bone level changes, a statistically significant decrease was observed in bone level in the immediate loading group compared with the conventional loading group (weighted mean difference [WMD] = −0.127; 95% CI, (−0.195, −0.059); *P* < 0.00001).

**Conclusion:**

Lower implant survival rates and a decrease in marginal bone level was observed compared to immediate loading with conventional loading.

## Introduction

Since Brandmark’s comparative study (1960s) found osseointegration theory, clinical implant dentistry has developed significantly [1]. In this context, the role of implant-supported removable prostheses has received increased attention. Compared with conventional removable prostheses, implant-supported prostheses will dramatically improve patients’ satisfaction and quality of life [2]. Compared with implant-supported fixed prostheses, implant-supported removable prostheses are more conducive to daily cleaning [3].

Conventionally, an unloaded healing period after implant placement is necessary to promote osseointegration, avoid soft tissue encapsulation, and improve implant survival [4]. Generally, the unloaded healing period is 3 months for the mandible, and 6 months for the maxilla. However, patients are usually limited to wearing aesthetically inferior and functionally inferior provisional prostheses in the time interval between implant placement and restoration finalisation. To provide patients with earlier aesthetic and functional recovery, many studies have been conducted in recent years to compare the effects of immediate loading (IL) durations on implant survival, changes in bone levels, and other aspects [5–8].

To date, several systematic evaluations and meta-analysis studies have attempted to compare the effects of IL and conventional loading (CL) on implant-supported dentures. However, the article by Alsabeeha et al. [9] included only mandibular overdentures and analysed only the success rates. The article by Schimmel et al. [10] included both maxillary and mandibular dentures. However, the scope was still limited to full-maxillary dentures, and the results were limited to 1-year success rates. The article by Ye et al. [11] analysed success rates and included bone level changes, but the inclusion was limited to mandibular full-maxillary dentures.

To our knowledge, no study has meta-analysed the clinical outcomes of immediately loaded and conventionally loaded implant-supported removable dentures (including both full-maxillary dentures and partial removable dentures).

This systematic evaluation and meta-analysis aims to compare the difference in implant survival and bone level changes between immediately loaded and conventionally loaded implant-supported removable dentures (including both full-maxillary dentures and partial removable dentures), and suggests possible reasons for the difference.

## Materials and methods

This systematic review is reported according to the Preferred Reporting Items for Systematic Reviews [12]. Before initiating this study, the protocol was registered in PROSPERO (International Prospective Systematic Evaluation Registry) (CRD42023452314) on August 21, 2023. Also, in August 2023, we started the systematic review.

### PICOS questions

The systematic review of randomised clinical trials (RCTs) aimed to answer the following PICOS questions:

**Population (P)**: Patients undergoing implant-supported removable prostheses.

**Intervention (I):** Patients with immediate load after loading.

**Comparison (C):** Patients with conventional load after loading.

**Outcome (O):** The primary outcome was implant survival.

The secondary outcomes were bone level changes, masticatory efficiency, implant stability quotient (ISQ), and patient satisfaction.

**Study(S):** RCTs were the inclusion condition for this meta-analysis.

According to the 6th European Association for Osseointegration (EAO) Consensus Conference 2021, the timing of loading was defined as follows:

- **Immediate loading (IL):** Within 7 days between dental implants and prosthesis loading.
- **Conventional loading (CL):** Over 2 months between dental implants and prosthesis loading.

### Search strategies

A search without language and time restrictions was undertaken in August 2023 in the following electronic databases: PubMed, Cochrane Library, Web of Science. The search strategy is shown in Table 1. Manual searching in different implant-related journals were as follows: *European Journal of Oral Implantology, The Journal of Oral Implantology, International Journal of Oral and Maxillofacial Surgery, Clinical Oral Implants Research, International Journal of Periodontics and Restorative Dentistry, Clinical Implant Dentistry and Related Research, International Journal of Implant Dentistry, Journal of Clinical Periodontology, Journal of Periodontology.* Online clinical trial information databases were also manually searched (clinicaltrials.gov).

**Table 1:** Including the method of the study.

Database	PubMed	Cochrane library	Web of science
**Population**	(((((((((((((((((((((Dental Prosthesis, Implant-Supported[MeSH Terms]) OR (Dental Prosthesis, Implant Supported[Title/Abstract])) OR (Implant-Supported Dental Prosthesis[Title/Abstract])) OR (Dental Prostheses, Implant-Supported[Title/Abstract])) OR (Implant Supported Dental Prosthesis[Title/Abstract])) OR (Implant-Supported Dental Prostheses[Title/Abstract])) OR (Prostheses, Implant-Supported Dental[Title/Abstract])) OR (Prosthesis, Implant-Supported Dental[Title/Abstract])) OR (Denture, Implant-Supported[Title/Abstract])) OR (Denture, Implant Supported[Title/Abstract])) OR (Implant-Supported Denture[Title/Abstract])) OR (Dentures, Implant-Supported[Title/Abstract])) OR (Implant Supported Denture[Title/Abstract])) OR (Implant-Supported Dentures[Title/Abstract])) OR (Prosthesis Dental, Implant-Supported[Title/Abstract])) OR (Dental, Implant-Supported Prosthesis[Title/Abstract])) OR (Dentals, Implant-Supported Prosthesis[Title/Abstract])) OR (Implant-Supported Prosthesis Dental[Title/Abstract])) OR (Implant-Supported Prosthesis Dentals[Title/Abstract])) OR (Prosthesis Dental, Implant Supported[Title/Abstract])) OR (Prosthesis Dentals, Implant-Supported[Title/Abstract]))	(MeSH descriptor: [Dental Prosthesis, Implant-Supported] explode all trees) or ((Dentals, Implant-Supported Prosthesis or Implant-Supported Prosthesis Dental or Implant-Supported Denture or Implant-Supported Dental Prosthesis or Prostheses, Implant-Supported Dental or Implant Supported Dental Prosthesis or Prosthesis Dental, Implant Supported or Denture, Implant Supported or Prosthesis Dentals, Implant-Supported or Implant Supported Denture or Implant-Supported Dental Prostheses or Prosthesis, Implant-Supported Dental or Dental, Implant-Supported Prosthesis or Prosthesis Dental, Implant- Supported or Denture, Implant-Supported or Implant-Supported Dentures or Dental Prosthesis, Implant Supported or Dentures, Implant-Supported or Dental Prostheses, Implant-Supported or Implant-Supported Prosthesis Dentals):ti,ab,kw)	TS=(Implant-Supported Dental Prosthesis OR Implant Supported Dental Prosthesis OR Implant- Supported Dental Prostheses OR Implant- Supported Denture OR Implant Supported Denture OR Implant-Supported Dentures OR Implant-Supported Prosthesis Dental OR Implant- Supported Prosthesis denials)
**Intervention**	((((((((((Immediate Dental Implant Loading[MeSH Terms]) OR (Dental Implant Loading, Immediate[Title/Abstract])) OR (Socket Shield Technique[Title/Abstract])) OR (Socket Shield Techniques[Title/Abstract])) OR (Technique, Socket Shield[Title/Abstract])) OR (Socket-Shield Technique[Title/Abstract])) OR (Socket-Shield Techniques[Title/Abstract])) OR (Technique, Socket-Shield[Title/Abstract])) OR (Early Dental Implant Loading[Title/Abstract])) OR (Dental Implant Loading, Early[Title/Abstract])))	(MeSH descriptor: [Immediate Dental Implant Loading] explode all trees) or ((Early Dental Implant Loading or Dental Implant Loading, Early or Technique, Socket-Shield or Socket- Shield Techniques or Socket-Shield Technique or Technique, Socket Shield or Socket Shield Technique or Socket Shield Techniques or Dental Implant Loading, Immediate):ti,ab,kw)	TS=(Immediate Dental Implant Loading OR Socket Shield Technique OR Socket Shield Techniques OR Socket-Shield Technique OR Socket-Shield Techniques OR Early Dental Implant Loading)

### Inclusion and exclusion criteria

The inclusion criteria are as follows: RCTs with open or blinded outcomes assessment; patients with implant-supported removable prostheses; patients divided into IL and CL; and at least 15 participants.

Animal studies, studies with different implants between groups, and zygomatic implants were excluded. Case reports, review studies, and meeting abstracts were also excluded.

### Data collection and management

Two reviewers (ZW, HC) independently screened the titles and abstracts of studies identified from the electronic searches and hand-searching. When there was disagreement, a third reviewer (SL) made the final decision.

Two reviewers (ZW, HC) made the final decisions based on the complete reports of studies identified during titles and abstracts screening. Disagreements were resolved by discussion with a third reviewer (SL).

### Quality assessment and risk of bias

Two authors (ZW and HC) independently assessed the risk of bias for the included studies. The revised Cochrane risk-of-bias tool for randomised trials (RoB 2) [13] was used to determine the included RCTs. Six criteria were evaluated: selection bias, performance bias, detection bias, attrition bias, reporting bias, and other bias.

When faced with disagreements, a third reviewer will join to resolve them by discussion. No study was excluded because of the risk of bias within a study. The overall bias of RCTs is judged as follows:

- **Low risk of bias:** No high risk in the above criteria.
- **Moderate risk of bias:** Only one high risk in the above criteria.
- **High risk of bias:** More than two high risks in the above criteria.

### Data extraction and meta-analysis

The finally included studies extracted the following data: country of the study, number of patients, number of implants, patient’s age, prostheses type, gender of the patient, CL and IL time, follow-up visits, prostheses position, number of survival implants, bone level change, prostheses complications, and masticatory performance. Only the primary factors were meta-analysed because the secondary factors had different test conditions and outcome indicators.

Forest plots were used to present the results, displaying the mean differences with a 95% confidence interval (CI) between the IL and CL groups. The implant survival rate was evaluated as a binary variable outcome. The bone level changes were weighted mean difference (WMD) for continuous outcomes accompanied by 95% CIs. *I*^2^ statistics were used to express the percentage of the total variation across studies because of heterogeneity. When *I*^2^ > 75%, the study is considered high risk; and when *I*^2^ <25%, the study is regarded as low risk. The random-effect model is used when there is a significant heterogeneity (*I*^2^ > 50%)15 between the test and control study. A funnel plot is drawn to show the main biases related to sample size. When the number of meta-analyses published is more than 10, Begg’s tests will be used to test publication bias.

The data were analysed using the statistical software and statistical software programme (STATA-12; Stata Corp LP) and Review Manager (version 5.2.8).

### Quality of evidence and confidence in results

The Grading of Recommendations Assessment, Development, and Evaluation (GRADE) methodology was used to assess the quality of evidence and to rate the quality of treatment effect estimates at different loading times. The confidence level of the evidence on implant survival and changes in bone tissue levels was assessed in a systematic evaluation and META analysis. Five domains were considered: overall risk of bias, directness of evidence, consistency of results, precision of estimates, and risk of publication bias. The quality of the evidence and the credibility of the results were categorised into four categories: high, medium, low, and very low.

## Results

### Literature search

**Figure 1:**
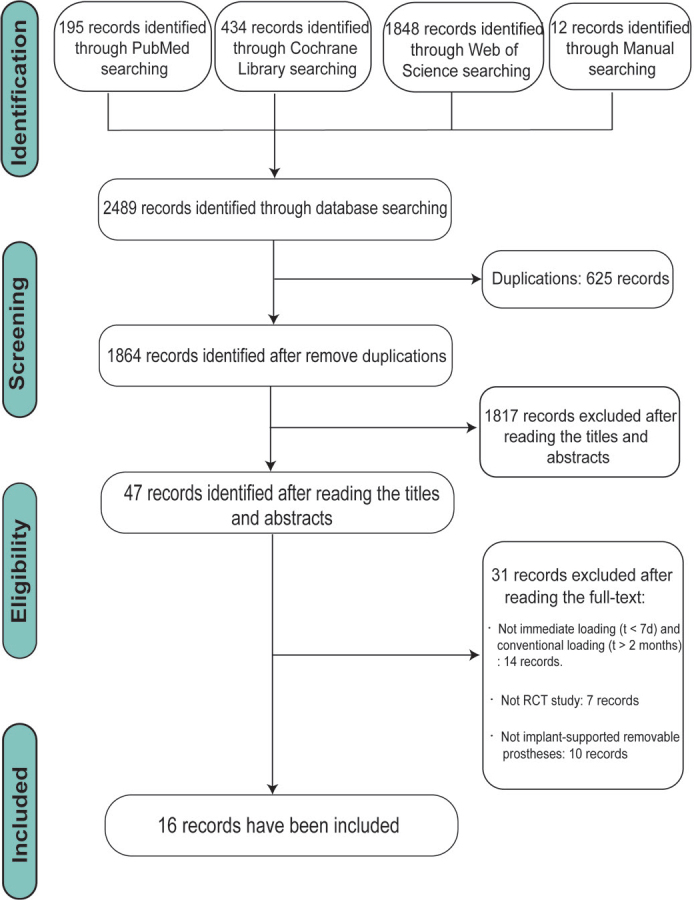
Flow diagram of studies identified, included, and excluded.

The database search and manual search identified 878 studies, including 195 from PubMed, 237 from Web of Science, 434 from The Cochrane Library, and 12 from manual searching. In all, 217 duplicates were removed. After evaluating the titles and abstracts of studies, 57 were selected for full-text assessment. After the studies had been read, 31 were excluded, and 16 publications were included in this review (Figure 1) (agreement = 93.4%; kappa = 0.73). All the included studies were RCT, and all patients with implant-supported removable prostheses were divided into IL and CL groups.

### Characteristics of including studies

Table 2 shows the characteristics of the included studies. The 16 studies [14, 14–28] (10 trials) finally selected included 543 participants with 1595 implants. Of these 16 studies, 2 were split-mouth trials, and the other 14 were parallel. Moreover, 805 implants were assigned to the IL group, and 790 were assigned to the CL group. All the experiments showed that the loading time of the IL group was within 7 days after operation (the shortest was intra-operative; the longest was 1 day after operation), while the loading time of the CL group was over 2 months after operation.

**Table 2:** Characteristics of included studies.

Reference	Country	Patients (implant) Immediate/conventional	Mean age (years) Immediate/conventional	Study design (RCT)	Prosthese type	Gender (male/female)	Immediate loading (after operation)	conventional loading (after operation)	Follow-up visits	Prosthese position	Experience of surgical operators	Missing implants (patients) (immediate/conventional)
Komagamine et al. (2019);	Japan	10(20)/ 9(18)	69.2 ± 10.6 /66.6 ± 9.1	Parallel	Two-implant overdentures retained by magnet attachments	9/10	Within 24 hours	3 months	1, 3, 6, 12, 24, 36 months after implant surgery	NM	Experienced implantologist	0/1(1)
Omura et al. (2016)									36, 48, 60 months after implant surgery			
Katheng et al. (2021)									4, 24 months after loading			
Abou-Ayash et al. (2020)												
Mundt et al. (2017)	Germany	81(81)/ 77(77)	70.4 ± 5.9 / 69.1 ± 6.4	Parallel	Complete denture on a single midline implant	92/66	Within 24 hours	3 months	1, 2, 3, 7 days after implant surgery ; 3 months after loading in immediate group 1, 4, 7 months after loading	Mandibular midline	Experienced clinicians	9(9)/1(1)
Schwindling et al. (2018)												
AlJaghsi et al.(2021);	Germany	38(100)/38(100)	66.4 / 65.4	Parallel	Removable partial dentures on mini-implants	29/47	24-48 hours	4 months	0.5, 4, 4.5, 12, 24, 36 months after implant surgery	Depending on the patient’s condition	Experienced dentists	8(3)/5(2)
Mundt et al. (2023)												
Daher et al. (2021)	Italy	18(60/60)	50 (only mean age)	Split mouth	Removable partial dentures	NM	Within 48 hours	3-3.5 months	0, 3-3.5, 15 months after implant surgery	Maxillary bilateral posterior/ posterior to the canines	Experienced oral surgeon	NM
Elsyad et al. (2014)	Egypt	18(32)/18(34)	60.3 ± 5.01 / 58.9 ± 6.6	Parallel	Locator-retained mandibular overdenture	20/16	Within 24 hours	3 months	0, 6, 12 months after loading	Canine region of the mandible	Oral surgeon	2(1)/0
Salman et al. (2019)	America	11(22)/12(24)	70.9 / 69.4	Parallel	Overdentures	12/11	Immediate	3 months	6, 12, 60 months after implant surgery	Canine/lateral incisor position	Experienced operator	NM
Schincaglia et al. (2016)	America	16(32)/15(30)	66.6 ± 10.2/ 66.2 ± 8.6	Parallel	Locator-Retained Mandibular Overdenture	20/12	Immediate	3 months	1, 2, 12, 24, 52 weeks after implant surgery	Mandibular	Experienced operator	2(1)/0
Schuster et al. (2020)	Brazil	10(20)/10(20)	66.8 / 67	Parallel	Implant-retained mandibular overdenture	8/12	Immediate	3 months	3, 6, 12 months after implant surgery ; 1,3,12 months after loading	Mandibular mental foramina region	Experienced surgeon	2(3)/ 2(2)
Giannakop oulos et al. (2017)	Germany	25(50/50)	69.42 (only mean age)	Split mouth	Overdentures on implants loaded with locator-analog attachments.	24/28	Within 24 hours	3 months	3, 6, 9, 12 month after loading	The regions of the second incisors or canines and the first or second premolars	Specialized dentists	1(1)/1
Passia et al. (2022)	Germany	50(50)/51(51)	60-89 (only age range)	Parallel	Overdentures on single implants	NM	24-48 hours	3 months	1, 4, 12, 24, 60 month after loading	Anterior mandible	NM	9(9)/2(2)
Elsyad et al. (2012)	Egypt	18(36)/18(36)	63.2 ± 2.69 / 64.6 ± 3.01	Parallel	Two implants supporting a ball-retained mandibular overdenture	22/14	Within 24 hours	3 months	4, 12, 36 months after implant surgery	Canine area	Prosthodontist	2(2)/0

As for the follow-up period, the maximum period was 60 months, and the minimum was 7 days 20. There were five implant-supported removable prostheses types: two-implant overdentures (four studies) [17–19, 28], one-implant overdentures (four studies) [20–22, 27], locator-retained overdenture (three studies) [24, 26, 29], removable partial dentures (three studies) [14, 15, 29], and two studies did not mention. Experienced surgeons performed all procedures except one study [27] that did not mention the surgeon’s specialty.

### Quality assessment and risk of bias

**Figure 2:**
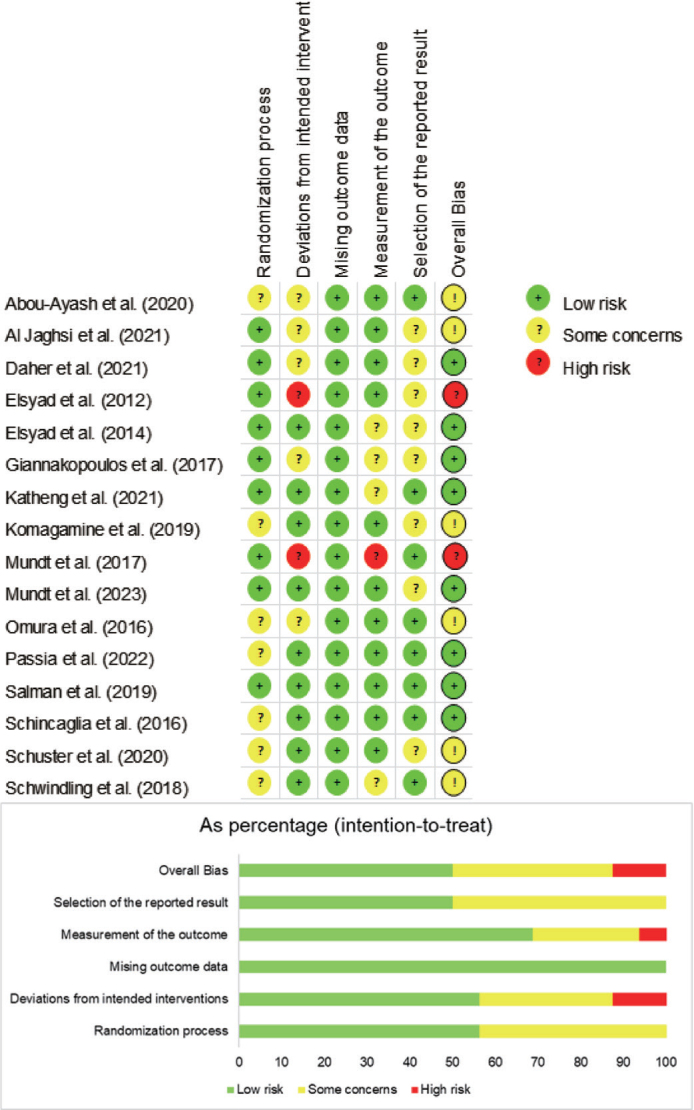
Risk of bias summary for selected randomised controlled trials.

Figure 2 depicts the bias assessment for the included studies using the RoB 2 framework. Eight studies showed a low risk of bias, while six raised some concerns. Also, two studies were at high risk because of considerations about the outcome measures and deviation from the intended intervention.

### Meta-analysis

**Figure 3:**
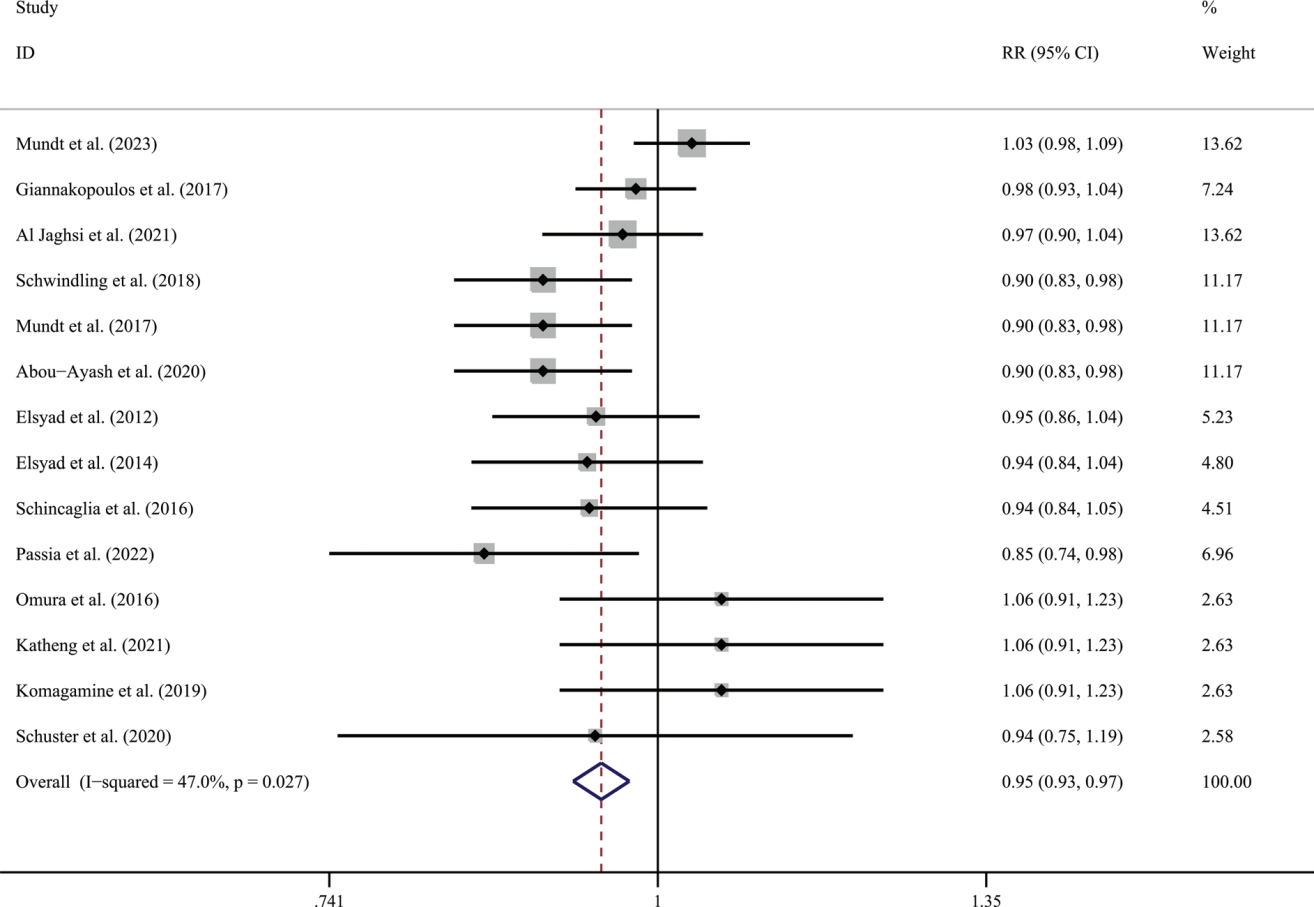
Forest plot of implant survival rate compared with delayed loading for the implant as the statistical unit.

**Figure 4:**
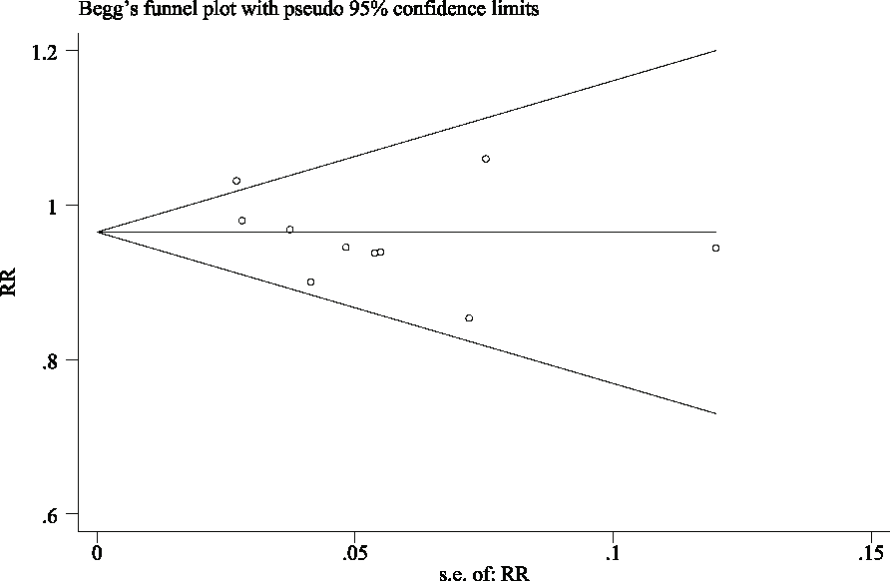
Funnel plot illustrating meta-analysis of implant survival rate compared with delayed loading for the implant as the statistical unit.

Fourteen studies reported an implant survival rate (976 patients and 1429 implants), which had three studies (1 trial) [17, 18, 20] reporting that the IL group had a 100% survival rate, and three studies (3 trials) [24, 26, 28] reported that the CL group had a 100% survival rate. Regarding implant numbers as statistical units to make a meta-analysis for these studies, the mean survival rates were 97.5% in the CL group and 93.0% in the IL group. The meta-analysis came to a statistically significant lower survival rate for the IL group than that in the conventional group (RR = 0.950; 95% CI, (0.926, 0.974); *P* = 0.027; *I*² = 47%) (Figure 3). Detecting by the Begg test, there was no publication bias for the above studies (*P* = 0.661; Figure 4).

**Figure 5:**
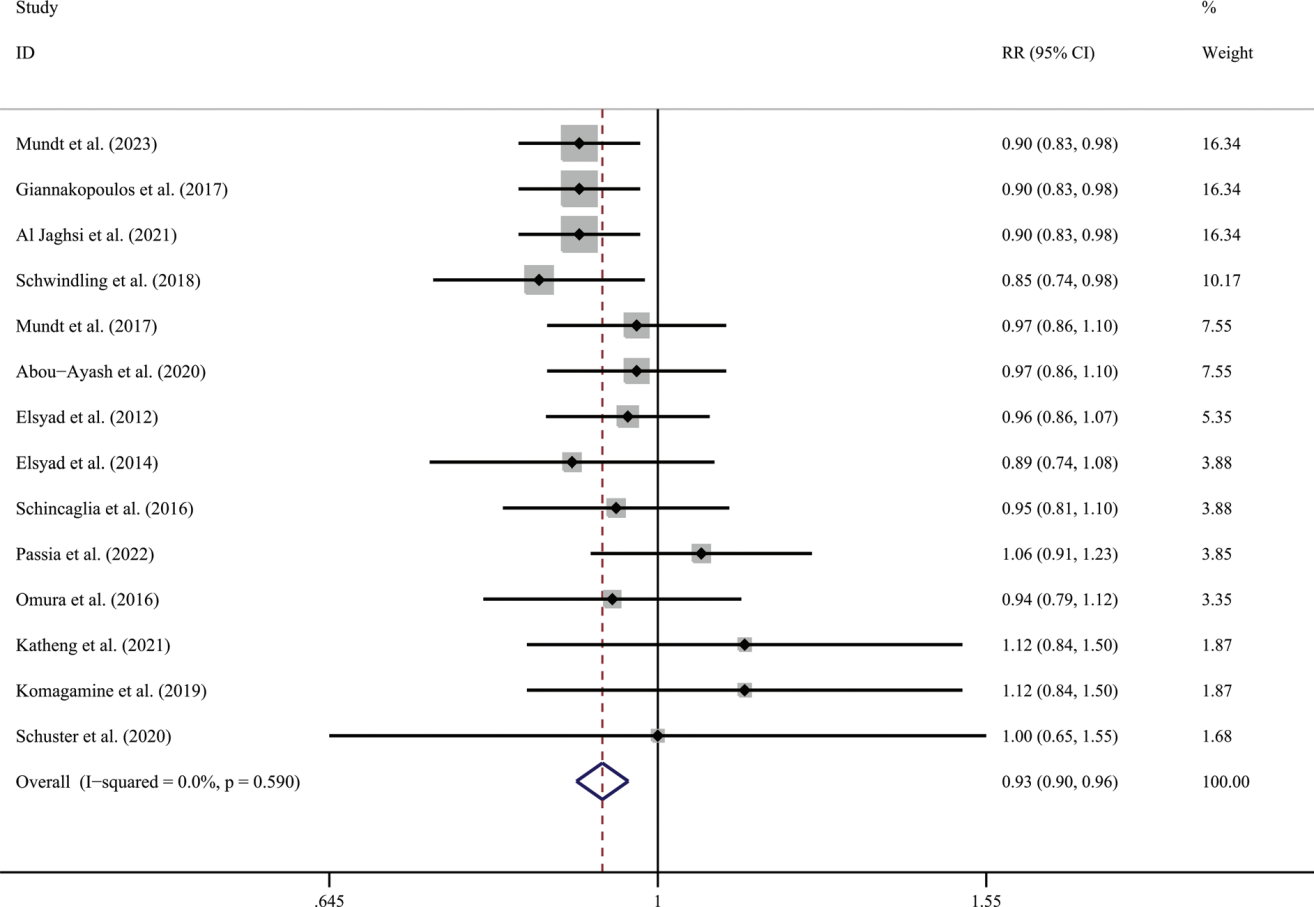
Forest plot of implant survival rate compared with delayed loading for the patient as the statistical unit.

**Figure 6:**
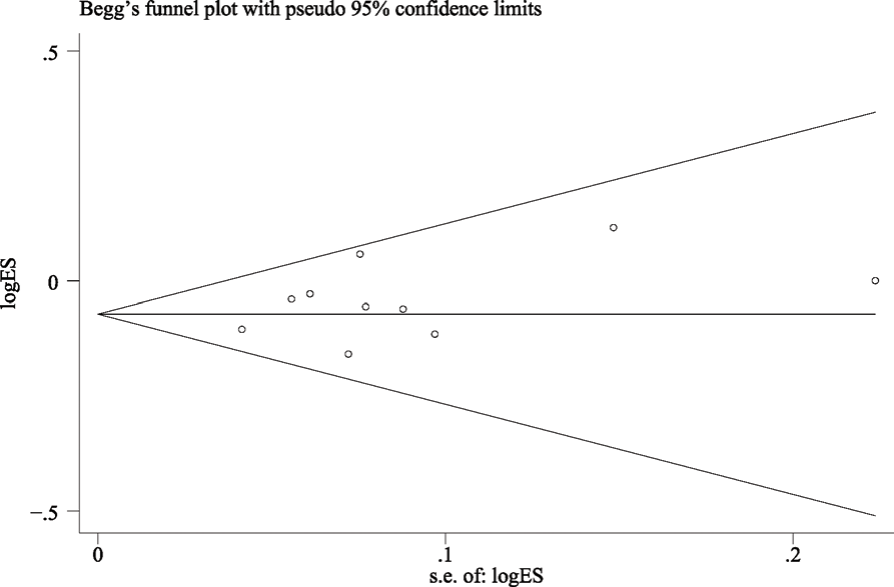
Funnel plot illustrating meta-analysis of implant survival rate compared with delayed loading for the patient as the statistical unit.

Regarding patients as statistical units to make a meta-analysis for these studies, the mean survival rates were 97.1% in the CL group and 90.1% in the IL group. The meta-analysis came to a statistically significant lower survival rate for the IL group when compared with the conventional group (RR = 0.929; 95% CI, (0.897, 0.961); *P* = 0.590; *I*² = 0%) (Figure 5). Detecting by the Begg test, there was no publication bias for the above studies (*P* = 0.139; Figure 6).

**Figure 7:**
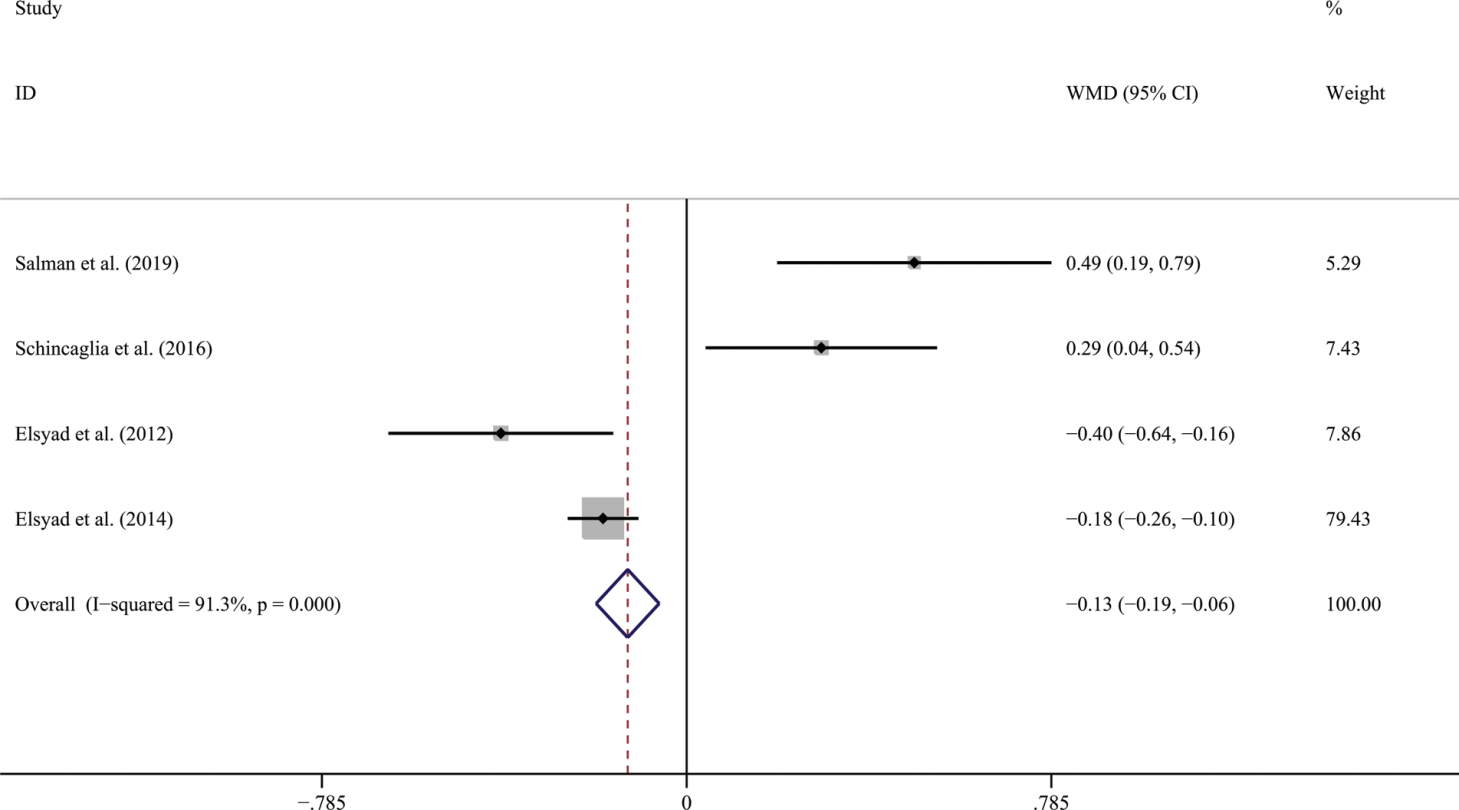
Forest plot of bone level change compared with delayed loading.

**Figure 8:**
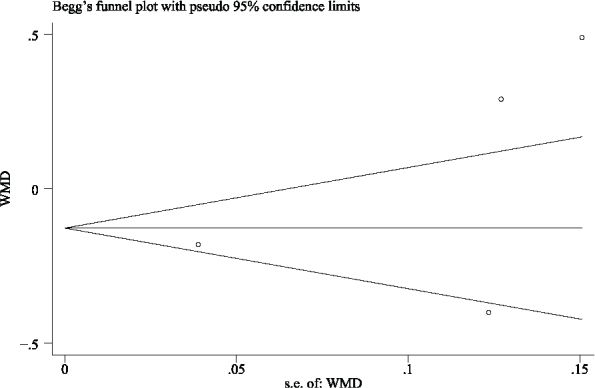
Funnel plot illustrating meta-analysis of bone level changes compared with delayed loading.

Five studies (286 implants) reported the bone level change; four of them [24–26, 28] reported the mean of changes, but another one [16] only reported the range of bone tissue changes, and then the studies that reported the range of age were excluded from the meta-analysis. Among the five included studies, the longest follow-up observation time for bone tissue level changes was 60 months, and the shortest was 6 months. Since all included studies were observed at 12 months, the bone level changes at 12 months were extracted for meta-analysis. The result shows significant differences in bone level changes between the CL and IL groups (WMD = −0.127; 95% CI, (−0.195, −0.059; *P* < 0.00001) (Figure 7). Random effects model was used because *I*² > 50%. No publication bias could be seen from the funnel plot (Figure 8). Since the number of pieces of literature included is less than 10, the Begg test was not used.

### Quality of evidence

**Figure 9:**
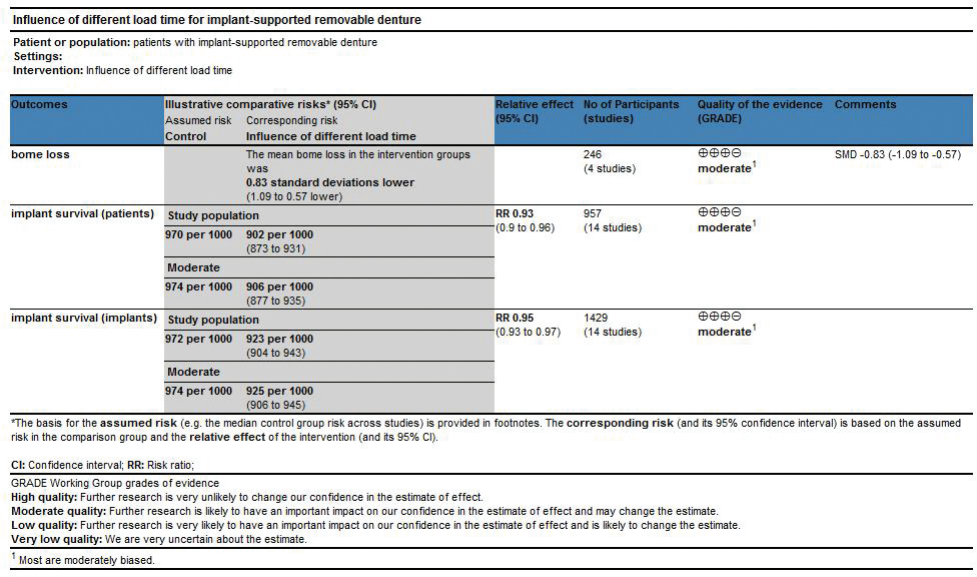
Summary chart of findings.

Figure 9 shows the quality of evidence and confidence in the results. The quality of evidence for comparing the IL and CL groups was moderate regarding implant survival and change in implant-bone levels. The main reason for the downgrade was the high number of medium risks of bias in the included studies.

## Discussion

### Principal findings

This systematic review included 16 studies (10 RCTs), with 543 patients and 1595 implants, that compared IL versus CL in implant-supported removable prostheses. There is a statistically significant lower survival rate for the IL group than the CL group, and whether to use the implant as unit or patient as unit. It suggests that implants with IL have a lower survival rate than CL on implant-supported removable prostheses. At the time of analysis of the included studies, implant loss within 6 months accounted for 71.4% of all loosed implants. At the same time, studies have shown that lower initial stability does not adversely affect implant survival and marginal bone levels under no load [30]. The lack of initial stability may be because of a lower survival rate [31, 32]. Many studies suggest improving implant materials, design, and flapless surgery to improve initial implant stability [31, 33, 34]. It has been reported that IL did not result in a statistically significant decrease in implant survival when initial stability was maintained [23, 35]. Many other elements might influence the implant survival rate. We also analysed peri-implant bone loss and found that IL had a statistically significant higher bone loss than CL.

Five studies have reported masticatory performances: two used chewing gum and gummy jelly [16, 18] two used standardised test food (Optical) to test [16, 27], and the final one used two-layered chewing gum [36]. The study that used two-layered chewing gum reported that the chewing gum’s colour-mixing ability was better in the IL group than in the CL group. One study reported the IL group exhibited a higher gummy jelly score than the CL group. However, there was no significant difference between the IL and CL groups in masticatory efficiency. For the implant stability coefficient (ISQ), Mundt [15] reported that different loading methods had no difference in ISQ, and Daher [22] reported that the IL group had a significantly lower ISQ than the CL group at the beginning of implantation. There was no significant difference after 6 months. Elsyad [24] reported that the ISQ of the IL group was significantly lower than that of the CL group at the beginning of loading. In comparison, the ISQ of the IL group was substantially higher than that of the CL group after 6 months of loading. After 12 months of loading, there was no difference between the two groups. As for patient satisfaction, Omura [18] and Al Jaghsi [14] reported higher early satisfaction in the IL group, while Abou-Ayash [19] reported no difference in patient satisfaction between the CL and IL groups. No meta-analysis was performed because the test conditions and secondary factors’ outcome measures differed.

### Agreements and disagreements with previous systematic reviews

Previous studies have examined the effect of loading time on implants. However, this is the first analysis to compare the efficacy of loading time on removable dentures.

Ebrahim [37] reported in 2022 that CL had higher implant survival when both fixed and removable dentures were included. There are no significant differences in implant survival and bone loss between immediate and CL when limiting the scope of the study to fixed dentures [2, 35]. But Chen reported in 2019 that CL had higher implant survival when focussed on fixed dentures. According to CL or IL methods, marginal bone loss of implants was compared in a systematic review published by Liu in 2022 [38]. Therefore, statistically different implant survival rates and bone level changes under different loading methods for removable dentures are reasonable.

### Clinical implications

The present systematic review showed IL had higher implant failure rates and peri-implant bone loss than CL when implant-supported removable dentures were studied. However, the survival rate of immediately loaded implants will be significantly improved if the initial implant stability is enhanced by optimising implant materials and designs, implant surgical methods, among others. Therefore, we believe that under the premise of ensuring initial stability, immediate load can be considered to make the patient’s aesthetics and function recover early.

Implant restoration is not determined solely by the loading pattern of the upper prosthesis, and clinicians should consider the patient’s specific conditions [39, 39, 40].

## Limitations and recommendations for future research

In reconstructing the implant-supported removable partial prosthesis, the loading method affects implant survival rate and peri-implant bone level, patient satisfaction [6, 41], masticatory efficiency [42, 43], and so on. However, insufficient controlled studies and consistent measurement standards provide sufficient data for a meta-analysis of these secondary outcomes. Well-designed RCTs with consistent criteria are needed to analyse these important clinical outcomes. At the same time, the variable length of follow-up included in the studies was a limiting factor.

## Conclusions

Based on the meta-analysis and systematic review of the above RCTs, the following conclusions were drawn:

- The meta-analysis showed a statistically significantly lower survival rate for the IL group than the CL group.
- There was a statistically significant difference in bone level changes when immediate loading was compared with CL.
- As to other secondary outcomes, we need more high-quality RCTs to determine the difference.
